# Doctor Bacon’s Case

**Published:** 1858-03

**Authors:** W. H. Byford


### DOCTOR BACON'S CASE.

**Died, Feb. 17th, 1858, at his residence, of Bright's disease, Milton Bacon, M. D., of Lake County, Illinois, aged 43 years.**

The profession, and the whole neighborhood of Dr. Bacon's residence, have sustained an irreparable loss in his death. His education was not *finished* in the college hall, for, although in early life he improved to the utmost of his abilities the ample advantages afforded by the best medical colleges, and graduated with much credit, he has been progressive in his attainments, and when he died was fully up with the improvement of the present time in the profession. A man of unexceptionable moral character and ethical propriety, his consultation practice was large, and all his advice was fraught with that soundness of judgment and discriminating acuteness which vast professional knowledge and extensive experience only can insure; while the confidence inspired by his conscientious and earnest demeanor, was of great service to his patients and medical associates. His friends and bereaved family can but derive consolation in the general condolence of his entire acquaintance. The mysterious working of Providence is never more profound and inscrutable than in such dispensations as the removal of men like Dr. Bacon, whose mental maturity, fulness of scientific attainments and ripeness of experience, the fruition of a long life of laborious and severe toil, render them invaluable to suffering humanity.

By request of his medical men, I subjoin the following relation of his case. W. H. Byford.

I was called to see Dr. Bacon, in consultation with Drs. Cory and Bullock, of Waukegan. The history, as related to me by himself, Mrs. B., and the medical gentlemen present, was as follows: He had been practising medicine for about twenty years in the neighborhood where he then lived. It is a high, broken, healthy lake coast, six miles north-west from Waukegan. Up to July last, he had enjoyed uninterrupted good health, and was regarded as one of the most hale members of the medical profession in that vicinity. Rather short in stature, portly,—in fact, corpulent in form, he was also jocund and lively in spirit. One peculiarity, which is said to have been observed by him since his earliest recollection, was an irregularity of the pulse—an omission of about one beat in twenty—while the heart was acting with regularity. He also assured me, that before July there was no irregularity of the heart's action of which he was conscious. For a short time prior to July, or early in the summer of 1857, he noticed slight œdema of the extremities, but it was evanescent in duration and partial in extent, so that it was not a source of much uneasiness to him. In July, while engaged in the active discharge of his professional duties, he was attacked with what was considered sun-stroke, from which he gradually recovered, with a slight paralysis of the whole left side as a sequel. This paralysis pretty rapidly declined, and in the course of a month seemed entirely gone, but returned in October for a few weeks. So soon as he had recovered from the sun-stroke, he was afflicted with indigestion, severe pain in the stomach, nausea and vomiting. Consentaneous with the dyspeptic symptoms, protracted and easily-excited paroxysms of palpitations of the heart supervened. These two symptoms became much more tolerable in a few weeks, but did not entirely subside. The functional irregularity of the heart was considered to be associated as an effect with the stomach derangement. Toward the latter part of his sickness, Dr. B. thought his heart was structurally diseased. There was no febrile movement of any consequence during the disease. Emaciation and debility, with drooping spirits, induced him to take a trip to the State of New York for his health, expecting to visit and spend some time at some of the favorite eastern watering places; but, after remaining with his friends for several weeks without benefit, he returned to the west. He continued to suffer from indigestion, palpitation, increasing emaciation and debility, without any other marked symptoms, until about two weeks before his death, when the left lower extremity became painful and swollen, and in a short time assumed an appearance resembling phlegmasia dolens. Cough and bloody expectoration began about a week before his death. The cough was very annoying, and often occurred in lengthened paroxysms, but more frequently a mouthful of almost pure blood was thrown up without much effort. His condition, when I saw him, was represented by the following array of symptoms, namely: general but moderate œdema, greatest in the lower extremities, particularly the left; surface quite perceptibly icteroid; temperature natural; breathing rapid, but not very laborious; frequent cough, and copious expectoration; pulse about 120 in the minute, feeble and intermittent; the intermission consisted in the omission of one pulsation about every twenty beats; otherwise, the pulse was regular. The pulsation of the heart could not be felt externally by the hand. Abdomen considerably enlarged by contained fluid. The liver was also greatly enlarged, extending across the epigastric into the left hypochondriac region, and down into the umbilical and lumbar region of the right side. His mind was quite clear, and he manifested an intelligent interest in the microscopic and chemical examinations. Upon auscultating the chest, the whole right lung, and much of the lower portion of the left, gave out the subcrepitant rale, indicating the occupancy of the small bronchia by the blood which was constantly being thrown from the lungs. The sounds of the heart were very feeble and distant, but not abnormal in quality, so far as I could discern with the imperfect examination I could make at the time. The urine was very dark colored, resembling porter, and so thick as to retain the bubbles of air for some time. Heat and nitric acid both showed a moderate quantity of albumen, while the microscope revealed blood and pus corpuscles. He remained in this condition, except a gradually increased embarrassment of respiration and prostration, until 17th, when he died. Post-mortem on the 18th. All the serous cavities of the body contained a large quantity of serum. The tunica albuginea testis and scrotum were also distended with serum. The pericardium was very much distended, which accounts for the feeble sounds of the heart. The right lung was engorged from top to bottom with thick tenacious bloody mucous, and about two-thirds of the lower portion of the left was in the same condition. Their structure did not seem to have undergone any change. The heart was hypertrophied and dilated until, with the blood it contained, it weighed forty-three ounces. After being evacuated and washed clean, its weight was twenty-seven ounces. It was dilated until it contained six ounces of blood. The valves, auriculo-ventricular and ventriculo-arterial, were healthy, and every way natural as to size. The stomach and intestinal canal throughout were quite sound. The liver was very much enlarged, but, it was believed, from congestion. The gall bladder was largely distended, and its neighborhood was stained by, while the cellular tissue of the whole body was infiltrated with, bile. The most remarkable changes, however, in the abdominal organs were discovered in the kidneys. The left was hypertrophied to what seemed about one-third more than natural size, but not otherwise altered; the right was structurally changed. The pelvis was larger than ordinary, and at its lower end pouched down to the size of a small hen's egg, in which was contained dark pus, blood and urine. The contents of the organ, although of urinous odor, had very little the appearance of that fluid in other respects. The cortical substance was very much thinned, and in parts of the walls completely replaced by a hard condensed fibrous membrane, while all of this structure was more than ordinarily condensed, and showed in several places, under a magnifier, irregular depositions of fibrin. The tubular structure was obliterated entirely in many places, and there were carneous agglomerations all over it. The immediate cause of Dr. Bacon's death was, doubtless, the extensive congestion of the lungs. Many of the symptoms that occurred during the progress of the disease were directly referable to the affection of the kidneys, and a matter of no small importance would be decided if we could compute the whole of the damage arising from this source. My *opinion*, and I think it is borne out by the present state of pathological knowledge, is, that the kidneys were the first organs diseased, and that all the other phenomena are directly or indirectly attributable to their functional improprieties. The essential condition of the kidneys, to the production of all the symptoms which constitute Bright's disease, or albuminuria, is an obstruction of a part or a whole of capillary tissues in which secretion takes place, so as to prevent them from performing their secretory functions, and cause the effusion of albumen from the blood. This may be and often is effected by fibrinous or albuminous infiltration. The deposit or infiltration may be the result of a well-marked chronic inflammation, as I think it was in the case of Dr. B., or a solid effusion from a less active condition of the circulation. The succession of symptoms, I think, bears me out in my assertion as to the agency of the kidneys. The Doctor spoke of having observed an evanescent œdema, but distinctly marked, before he was attacked with the train of symptoms which continued until his death. Next in order were the nervous system, sun-stroke, followed by partial paralysis. So soon as he recovered from the urgent head symptoms, painful dyspepsia and palpitation of the heart followed, and continued with varying gravity until the termination of the case in death. This is just the course we might expect to observe in cases of albuminuria. Heart affection is almost if not quite an essential element of Bright's disease; and the weight of authority inclines to the opinion, that it is one of the *effects* of albuminuria. Be this as it may, we have all the anatomical alterations and symptomatic manifestations necessary to make a complete case of this fatal malady. One rather singular circumstance is, that he had never complained of pain in the region of the kidneys, notwithstanding the amount of disease found in them after death. This unfortunate combination of organic disease rendered all but palliative treatment entirely profitless; and if the precise condition of his entire organism could have been disclosed months before his decease, the only result that did not obtain in the case, would have been an adverse and hopeless prognosis. The medical gentlemen, who attended him during his long illness with the devotion of true and worthy professional brethren, Drs. Cory, Bullock and Evans, of Waukegan, acting often in the capacity of watchers and nurses, have afforded to the profession an example worthy not only of all commendation but of imitation, and will be remembered with gratitude by the family and friends.

